# Optical imaging detects metabolic signatures associated with oocyte quality^†^

**DOI:** 10.1093/biolre/ioac145

**Published:** 2022-07-21

**Authors:** Tiffany C Y Tan, Hannah M Brown, Jeremy G Thompson, Sanam Mustafa, Kylie R Dunning

**Affiliations:** School of Biomedicine, Robinson Research Institute, The University of Adelaide, Adelaide, South Australia, Australia; Australian Research Council Centre of Excellence for Nanoscale Biophotonics, The University of Adelaide, Adelaide, South Australia, Australia; Institute for Photonics and Advanced Sensing, The University of Adelaide, Adelaide, South Australia, Australia; Victorian Heart Institute, Monash University, Clayton, Victoria, Australia; School of Biomedicine, Robinson Research Institute, The University of Adelaide, Adelaide, South Australia, Australia; Australian Research Council Centre of Excellence for Nanoscale Biophotonics, The University of Adelaide, Adelaide, South Australia, Australia; Institute for Photonics and Advanced Sensing, The University of Adelaide, Adelaide, South Australia, Australia; Fertilis Pty Ltd, Adelaide, South Australia, Australia; School of Biomedicine, Robinson Research Institute, The University of Adelaide, Adelaide, South Australia, Australia; Australian Research Council Centre of Excellence for Nanoscale Biophotonics, The University of Adelaide, Adelaide, South Australia, Australia; Institute for Photonics and Advanced Sensing, The University of Adelaide, Adelaide, South Australia, Australia; School of Biomedicine, Robinson Research Institute, The University of Adelaide, Adelaide, South Australia, Australia; Australian Research Council Centre of Excellence for Nanoscale Biophotonics, The University of Adelaide, Adelaide, South Australia, Australia; Institute for Photonics and Advanced Sensing, The University of Adelaide, Adelaide, South Australia, Australia

**Keywords:** autofluorescence, NAD(P)H, FAD, oocyte assessment, non-invasive, cellular metabolism, optical imaging, oxygen consumption rate, optical redox ratio

## Abstract

Oocyte developmental potential is intimately linked to metabolism. Existing approaches to measure metabolism in the cumulus oocyte complex (COC) do not provide information on the separate cumulus and oocyte compartments. Development of an assay that achieves this may lead to an accurate diagnostic for oocyte quality. Optical imaging of the autofluorescent cofactors reduced nicotinamide adenine dinucleotide (phosphate) [NAD(P)H] and flavin adenine dinucleotide (FAD) provides a spatially resolved indicator of metabolism via the optical redox ratio (FAD/[NAD(P)H + FAD]). This may provide an assessment of oocyte quality. Here, we determined whether the optical redox ratio is a robust methodology for measuring metabolism in the cumulus and oocyte compartments compared with oxygen consumption in the whole COC. We also determined whether optical imaging could detect metabolic differences associated with poor oocyte quality (etomoxir-treated). We used confocal microscopy to measure NAD(P)H and FAD, and extracellular flux to measure oxygen consumption. The optical redox ratio accurately reflected metabolism in the oocyte compartment when compared with oxygen consumption (whole COC). Etomoxir-treated COCs showed significantly lower levels of NAD(P)H and FAD compared to control. We further validated this approach using hyperspectral imaging, which is clinically compatible due to its low energy dose. This confirmed lower NAD(P)H and FAD in etomoxir-treated COCs. When comparing hyperspectral imaged vs non-imaged COCs, subsequent preimplantation development and post-transfer viability were comparable. Collectively, these results demonstrate that label-free optical imaging of metabolic cofactors is a safe and sensitive assay for measuring metabolism and has potential to assess oocyte developmental competence.

## Teaser

Optical imaging using confocal and hyperspectral microscopy is able to measure dynamic changes in oocyte metabolism.

## Introduction

Oocyte developmental competence; a term defined as the capability of the oocyte to resume meiosis, undergo fertilization and preimplantation embryo development, implant, and result in a healthy offspring [1]. The developmental competency of an embryo is highly dependent on the oocyte from which it is derived. Thus, selection of an oocyte with high developmental potential is a possible route to improve the success rate of in vitro fertilization (IVF) [2]. Morphological assessment of the oocyte remains the primary method of evaluation in the clinic, despite being subjective and inaccurate in predicting competency [2–5].

Much effort has been invested in understanding the metabolism of the cumulus oocyte complex (COC) [6–11] and how various metabolic pathways impact oocyte quality (for review, see [2]). Intra-oocyte ATP is an important determinant of oocyte quality, with a deficit in ATP associated with reduced developmental potential [12–14]. The oocyte relies predominantly on oxidative phosphorylation to generate mitochondrial-derived ATP, and via gap junctions, cumulus cells contribute to the pool of intra-oocyte ATP via glycolysis: a metabolic pathway not active in the oocyte [2, 15]. Many studies have attempted to find metabolic biomarkers associated with oocyte quality, including metabolites within follicle fluid [16], cumulus cell gene expression [1, 17, 18], and profiling of “spent medium” following in vitro maturation (IVM) [16, 19–22]. However, these methods have not been routinely implemented in the clinic. As the oocyte and cumulus cells utilize different metabolic pathways to generate intra-oocyte ATP, these approaches may fail to accurately predict oocyte quality as they measure metabolism of the whole COC and do not provide metabolic signatures of the separate cumulus and oocyte compartments [2]. Thus, development of a non-invasive assay that provides spatial information on metabolic activity in the COC may lead to an accurate diagnostic for oocyte quality.

Mitochondrial oxidative phosphorylation is the primary pathway for generating cellular ATP and is typically measured via oxygen consumption: the benchmark measurement in our field [23–26]. However, this measurement is unable to provide spatial information on individual cells where heterogeneity likely exists, as is the case for the COC [2]. To complement this, the recent use of label-free optical imaging of intracellular autofluorescence to non-invasively assess metabolism [27–30] is potentially very powerful, allowing for spatial information within the COC to be recorded. A large proportion of cellular autofluorescence is derived from the metabolic co-factors reduced nicotinamide adenine dinucleotide (NADH), reduced nicotinamide adenine dinucleotide phosphate (NADPH), and flavin adenine dinucleotide (FAD). Due to their near identical spectral properties, NADH and NADPH are collectively referred to as NAD(P)H [31]. These co-factors can be used to calculate the optical redox ratio (FAD / [FAD + NAD(P)H]), which has been used in other cell types as a measure of oxidative phosphorylation [32]. In the field of reproductive biology, optical imaging of these endogenous fluorophores using laser scanning confocal microscopy has been used to measure metabolism in oocytes and preimplantation embryos [33–36]. However, previous work has not assessed the accuracy of the optical redox ratio in measuring dynamic metabolic changes in the cumulus and oocyte compartments of the COC. This is particularly important as the captured fluorescence may include signatures from fluorophores other than FAD and NAD(P)H [27].

In this study, we assessed whether the optical redox ratio is a robust method to measure dynamic metabolic changes in the oocyte and cumulus cell compartments of the COC. We did this by comparing the optical redox ratio for each of the cell types with oxygen consumption of the whole COC. These were measured at basal levels and in response to modulators of oxidative phosphorylation. Oxygen consumption was measured using extracellular flux analysis which has been utilized in various cell types including cancer [37–39] and reproductive cell types [40–42]. We also determined whether label-free confocal imaging could detect metabolic differences in COCs with poor developmental potential. Following the demonstration that confocal imaging was robust and able to detect metabolic variance in COCs with poor developmental potential, we validated these results using a clinically appropriate imaging modality, namely hyperspectral microscopy. This requires a low energy dose, and thus has a very low potential for phototoxicity resulting from imaging. The safety of this imaging approach was examined by recording embryo development following IVF as well as postnatal outcomes following transfer of resultant embryos to recipients. In this study, we show the capacity of label-free optical imaging to separately measure metabolism in the oocyte and cumulus cell compartments of the intact COC, and the potential of this approach to safely assess oocyte developmental competence.

## Materials and methods

All reagents were purchased from Sigma Aldrich (St. Louis, MO, USA) unless stated otherwise.

### Animal ethics

Female (21–23 days) and male (6–8 weeks old) CBA × C57BL/6 first filial (F1) generation (CBAF1) as well as female (6–8 weeks old) Swiss mice were obtained from Laboratory Animal Services (LAS University of Adelaide, SA, Australia) and maintained on a 12 h light:12 h dark cycle with rodent chow and water provided ad libitum. All experiments were approved by the University of Adelaide’s Animal Ethics Committee and were conducted in accordance with the Australian Code of Practice for the Care and Use of Animals for Scientific Purposes.

### Media

All gamete and embryo culture took place in media overlaid with paraffin oil (Merck Group, Darmstadt, Germany) in a humidified atmosphere of 5% O_2_, 6% CO_2_ with a balance of N_2_ at 37°C unless stated otherwise. All media were pre-equilibrated for at least 4 h prior to use. All handling procedures were performed on microscopes fitted with warming stages calibrated to maintain the media in dishes at 37°C.

The base medium used for handling and culture was phenol red-free Minimum Essential Medium Eagle (MEM-E). For collection and imaging of immature COCs, ovaries were collected in handling medium comprised of MEM-E supplemented with 6 mM NaHCO_3_, 50 mg/L gentamicin sulfate, 10 mM HEPES, 2 mM glutamax (Gibco by Life Technologies, CA, USA), and 3 mg/mL fatty acid-free bovine serum albumin (BSA, MP Biomedicals, Albumin NZ, Auckland, NZ). The media was filtered before adding 1 × 10^−2^ μM estradiol [43, 44] and 50 μM 3-isobutyl-1-methylxanthine [45]. The handling medium for ovary collection and handling of COCs prior to IVM was filtered MEM-E supplemented with 6 mM NaHCO_3_, 50 mg/L gentamicin sulfate, 10 mM HEPES, 2 mM glutamax, and 5% (v/v) fetal calf serum (FCS) [46]. The culture medium for IVM was MEM-E supplemented with 26 mM NaHCO_3_, 50 mg/L gentamicin sulfate, 2 mM glutamax, 5% (v/v) FCS, 50 mIU/mL of recombinant human follicle-stimulating hormone (Los Angeles Biomedical Research Institute, LA, USA) and 3 ng/mL epidermal growth factor, hereafter referred to as “IVM medium” [9, 46, 47]. Research Wash Medium, Research Fertilization Medium and Research Cleave Medium (ART Lab Solutions, SA, Australia) supplemented with 4 mg/mL of low fatty acid BSA were used for IVF and embryo culture, respectively.

### Collection of immature cumulus oocyte complexes

Mice were injected subcutaneously with 5 IU equine chorionic gonadotrophin (eCG, Braeside, VIC, Australia). At 44 h post-eCG, mice were culled by cervical dislocation and ovaries collected in warmed handling medium. The COCs were isolated from ovaries by puncturing follicles using a 29-gauge × ½ in. insulin syringe with needle (Terumo Australia Pty Ltd., NSW, Australia). Isolated immature COCs were used for IVM or for imaging using two-channel laser scanning confocal microscopy to measure the optical redox ratio.

### In vitro maturation

Immature COCs were cultured in groups of 20 in drops of 100 μL IVM medium overlaid with paraffin oil. Mature COCs (12 h post IVM) were used for imaging using two-channel laser scanning confocal microscopy to measure the optical redox ratio. A separate cohort of COCs were matured in the absence or presence of etomoxir (100 μM; 16 h) [9]. Maturation of COCs occurred within 20% O_2_, 6% CO_2_ with a balance of N_2_ at 37°C. Following IVM, 12 h COCs were treated with metabolic inhibitors/uncoupler and imaged using two-channel scanning confocal microscopy. COCs matured for 16 h were either imaged (two-channel scanning confocal microscopy or hyperspectral microscopy) or fertilized in vitro.

### In vitro fertilization and embryo culture

One hour prior to IVF, male mice with proven fertility were culled by cervical dislocation with the epididymis and vas deferens collected in Research Wash Medium. Spermatozoa were released from the vas deferens and caudal region of the epididymis by blunt dissection in 1 mL of Research Fertilization Medium and allowed to capacitate for 1 h in a humidified atmosphere of 5% O_2_, 6% CO_2_ with a balance of N_2_ at 37°C. Mature COCs were then co-cultured with capacitated spermatozoa (35 000 sperm/mL) for 4 h at 37°C in a humidified atmosphere of 5% O_2_, 6% CO_2_ with a balance of N_2_ [48, 49]. The resulting presumptive zygotes were cultured in groups of 10–12 in 20 μL drops of Research Cleave Medium overlaid with paraffin oil in a humidified atmosphere of 5% O_2_, 6% CO_2_ with a balance of N_2_ at 37°C. Embryos were assessed for on-time morphological development on day 2 (2-cell; 24 h post-IVF) and day 5 (blastocyst-stage; 96 h post-IVF). The rate of development to the 2-cell and blastocyst stages was calculated from the initial number of oocytes. Two-cell-stage embryos were identified by the presence of two regular blastomeres of equal size, while blastocysts were identified by the presence of a blastocoel cavity ≥ two-thirds the size of the embryo; or expanded; or hatching.

### Use of metabolic inhibitors

Mitochondrial inhibitors (oligomycin, carbonyl-4-phenylhydrazone (FCCP) and Rotenone/antimycin A (Rot/AA)) used for this study were from the Seahorse XF Cell Mito Stress Test Kit (Agilent Technology, CA, USA). Inhibitors were dissolved as per manufacturer’s instructions in either MEM-E (for experiments where metabolic cofactors were measured by optical imaging) or Seahorse XF DMEM medium (measurement of oxygen consumption rate (OCR) by extracellular flux assay; Agilent Technology, CA, USA) and stored in −80°C. One hour prior to imaging or extracellular flux analysis, inhibitors were diluted to required concentrations with pre-warmed MEM-E or DMEM.

The optimal dose for each mitochondrial inhibitor/uncoupler was determined. Doses chosen for analysis (Supplementary Figure SS1) were based on manufacturers’ instructions and prior literature [41]. The final concentrations used for this study were 2.0 μM oligomycin; 1.0 μM FCCP, and 2.5 μM Rot/AA, as these elicited an appropriate response in oxygen consumption in COCs (Supplementary Figure SS1).

### Measurement of oxygen consumption rate in immature cumulus oocyte complexes using extracellular flux analysis

The base medium used for extracellular flux analysis was Seahorse XF DMEM medium, supplemented with 1 mM pyruvate (Agilent Technology, CA, USA), 2 mM glutamine (Agilent Technology, CA, USA) and 10 mM glucose (Agilent Technology, CA, USA). Immature COCs were isolated in pre-warmed handling medium as described above and placed into wells at a density of 20 COCs/well. One hour prior to the assay, COCs were washed three times in Seahorse XF DMEM before being replaced with fresh Seahorse XF DMEM and cultured for another hour in a non-CO_2_ gassed, humidified incubator at 37°C.

The Seahorse Bioscience XF analyzer and Mito Stress Test Kit (Agilent Technology, CA, USA) were used according to the manufacturer’s instructions. The sensor containing fluxpak (Agilent Technology, CA, USA) was hydrated and incubated overnight at 37°C in a non-CO_2_ gassed humidified incubator. The sensor containing fluxpak was calibrated for approximately 15 min as per manufacturer guidelines. Upon completion, the pre-warmed cell plate containing immature COCs was loaded into the machine. The OCR was analyzed using a protocol involving a 12 min equilibration period and alternating between a 3 min measurement period and a 3 min re-equilibration period. During the measurement period, the sensor containing the probe was lowered down, creating an airtight 2.3 μL microenvironment. The output of extracellular flux analysis was given as OCR in pmol/min/well.

In a separate cohort, the basal OCR was measured in immature COCs cultured in either Seahorse XF DMEM or MEM-E to ensure that the base medium did not affect oxygen consumption (Supplementary Figure SS2). This was performed as the goal of the study was to use optical imaging of autofluorescent metabolic co-factors to assess oocyte developmental competence and the need to culture COCs in standard IVM medium (MEM-E).

### Measurement of metabolic co-factors NAD(P)H and FAD using two-channel laser confocal microscopy

Immature or mature (12 h) COCs were imaged. Images were acquired at baseline (basal), followed by sequential exposure to oligomycin, FCCP and Rot/AA (15 min between each treatment). Following IVM in the absence or presence of etomoxir (16 h), mature COCs were imaged. Imaging of COCs occurred within a 35 mm glass-bottom dish (Ibidi, Martinsried, Planegg, Germany) in 2 μL of Research Wash Medium, overlaid with paraffin oil.

The autofluorescence intensity indicative of co-enzymes NAD(P)H and FAD content was recorded on an Olympus Fluoview FV10i confocal microscope (Olympus, Tokyo, Japan). Cells were excited at a wavelength of 405 nm (emission detection bandwidth: 420–450 nm) for NAD(P)H (referred to as the NAD(P)H channel), and excited at a wavelength of 473 nm (emission detection bandwidth: 490–590 nm) for FAD (referred to as the FAD channel) as described in ref [35]. Image acquisition occurred at 60× magnification, numerical aperture equal to *NA* = 1.4, with a single z-plane chosen for each COC where the oocyte diameter was largest. Imaging parameters were kept constant between replicate experiments. Fluorescence intensity was measured using ImageJ software (National Institute of Health). For oocyte intensity measurements, a region of interest was created that encompassed the entire oocyte for each COC. Quantification of intensity within the cumulus cell compartment was performed by creating two regions interest of equal size and placing these on opposing sides of, and adjacent to, the oocyte. A mean of these two regions of interest was calculated for each COC. The optical redox ratio was calculated using the intensity of the FAD channel divided by the sum of the intensity of NAD(P)H and FAD channels (FAD / (NAD(P)H + FAD), which reflects the activity of the mitochondrial electron transport chain and therefore indicates the dynamic changes of cellular metabolism [30].

### Hyperspectral autofluorescence and brightfield imaging

In this work, the hyperspectral microscopy system (Quantitative Pty Ltd, Mount Victoria, NSW, Australia) was built by adapting a standard epifluorescence microscope (Nikon Eclipse TiE, 40× objective, *NA* = 1.3), fitted with a multi-LED light source (Prizmatix Ltd, Givat-Shmuel, Israel). These low-power LEDs provided 56 spectral channels: 21 excitation wavelength ranges and 3 emission wavelength filters, covering excitation wavelengths from 348 to 649 nm and emission wavelengths from 450 to 715 nm (for details of spectral channels, see Supplementary Table SS1). The fluorescence of native endogenous fluorophores found within cells was captured by a 40× objective imaging onto a digital camera C1140, OCRA Flash 4.0 (Hamamatsu, Shizuoka, Japan) using all 56 spectral channels. Image acquisition times of up to 3 s per channel were used, with multiple averaging (typically 1–3 times) to optimize image quality and minimize any potential photo-damage to the cells in each channel.

Mature COCs were imaged on a microscope slide and mounted using a 0.12 mm Secure Seal spacer (Molecular Probes, Invitrogen). Blastocysts were imaged on glass bottom confocal dishes (Ibidi, Martinsried, Planegg, Germany) containing 2 μL of Research Wash Medium overlaid with paraffin oil. Hyperspectral microscopy images were taken by adjusting the input light beam to specifically focus on the equatorial plane of the oocyte for COCs (i.e., the widest diameter) or the inner cell mass for individual blastocysts.

### Analysis of hyperspectral microscopy data

To avoid errors in quantification, images were digitally processed first using the custom-made GUI_Preprocess v3.12 software [50]. This is required to remove image artifacts and noise, such as background fluorescence, Poisson's noise, dead or saturated pixels, and illumination curvature across the field of view, as described in detail in previous work [51–53]. At the beginning of each experiment, two calibration images were captured using the hyperspectral system: a “background” reference image of a culture dish with medium only, and another with calibration fluid only. The “background” reference image was included and subtracted from all images to remove any background signals. The microscope system was calibrated with a mixture of 50 μM NADH and 10 μM riboflavins whose spectrum spans all spectral channels and the concentration used sufficient for visualization of their autofluorescence. The excitation and emission spectra of this calibration fluid were measured using a spectrometer (FLS1000 Photoluminescence Spectrometer) and imaged on the hyperspectral microscope across all spectral channels.

Based on the known spectral properties of NAD(P)H and FAD [32, 54], we selected channels 1 and 2 (NAD(P)H channels) and channels 24 and 25 (FAD channels) (Supplementary Table SS1) to capture their autofluorescence. The COCs and blastocyst-stage embryos were manually segmented using the brightfield image to create a region of interest [49]. For COCs, the region of interest was as described above for confocal microscopy. For blastocyst-stage embryos, the region of interest was manually drawn around the inner cell mass. The intensity of NAD(P)H and FAD was quantified using custom-made GUI_Preprocess v3.12 software [50].

### Embryo transfer and postnatal outcomes

Mature COCs (16 h) were divided into two groups: those imaged using the hyperspectral microscope and those that were not. Both groups of COCs were then fertilized in vitro and allowed to develop to the blastocyst-stage. Embryos were assessed for on-time morphological development on day 2 (2-cell; 24 h post-IVF) and day 5 (blastocyst-stage; 96 h post-IVF). Resultant blastocyst-stage embryos were then vitrified. Embryo vitrification and warming were performed as previously described [49]. On the day of transfer, blastocyst-stage embryos were warmed 2 h prior to embryo transfer to allow sufficient time for recovery. Blastocyst-stage embryos were transferred into the uterine horns of pseudopregnant Swiss mice 2.5 days post-coitum. Embryo transfers were performed on mice under anesthesia with 1.5% isoflurane as previously described [49]. Eight to twelve morphologically normal, expanded blastocysts were transferred to each uterine horn. Mice were monitored daily. The number of pups for each recipient was recorded on delivery. Pregnancy rate was calculated from the number of pregnant recipients over the total number of pseudopregnant females. Live birth rate was calculated from the number of live pups over the number of embryos transferred (non-pregnant mice were excluded from this analysis). At post-natal day 21, offspring were weaned, accessed for gross facial deformities and weight recorded.

### Statistical analyses

Wave software (Agilent Technology, CA, USA) was used to determine OCR in pmol/min/well. Statistical analyses were carried out using GraphPad Prism Version 9 for Windows (GraphPad Holdings LLC, CA, USA) except for weight of offspring data where Statistical Package for Social Science (SPSS) version 28.0.1.0 software was used. Data were subjected to normality testing using the D’Agostino-Pearson Omnibus normality test prior to statistical analysis. Normally distributed data were analyzed either by an unpaired Student *t*-test or an ordinary one-way analysis of variance (ANOVA) with Holm-Šídák post-hoc test. For data that did not follow a normal distribution a Mann–Whitney test or Kruskal-Wallis test with Dunn post-hoc test were used. Continuous data are presented as mean ± SEM. Weight of offspring at weaning is presented as estimated marginal mean ± SEM and was analyzed by linear mixed model with litter size as a covariate. Categorical data are described as percentages and compared using the Fisher exact test. Details of statistical tests are stated in the figure legends. Statistical significance was set at a *P*-value <0.05.

## Results

We evaluated the robustness of the optical redox ratio in detecting dynamic metabolic changes in the oocyte and cumulus cell compartments of the COC by direct comparison with the OCR. Assessments were made at baseline (basal—no drug treatment) and following the sequential addition of mitochondrial inhibitors/uncoupler. These drugs act by inhibiting specific components of the electron transport chain (Supplementary Figure SS4) [55]. Oligomycin inhibits ATP synthase providing an indication of the proportion of oxygen used for ATP production. Thus, the OCR will decrease upon exposure to this compound. Based on its mode of action, we hypothesized that oligomycin exposure will lead to an increase in NAD(P)H, a decrease in FAD and thus, a decrease in optical redox ratio. FCCP is a mitochondrial uncoupler and acts by interfering with the proton gradient. This results in maximum oxygen consumption and as such, the OCR increases. We hypothesized that the addition of FCCP will lead to decreased NAD(P)H and increased FAD and optical redox ratio. Lastly, rotenone and antimycin A (Rot/AA) when added together block complexes I and III, respectively shutting down the electron transport chain entirely and decreasing the OCR. In this instance, we hypothesized that the addition of Rot/AA will lead to increased NAD(P)H, and decreased FAD and optical redox ratio. We tested these hypotheses in the oocyte and cumulus cell compartments of immature and mature COC.

###  

#### Optical redox ratio detects dynamic changes in immature COCs that correlate with oxygen consumption rate

**Figure 1:**
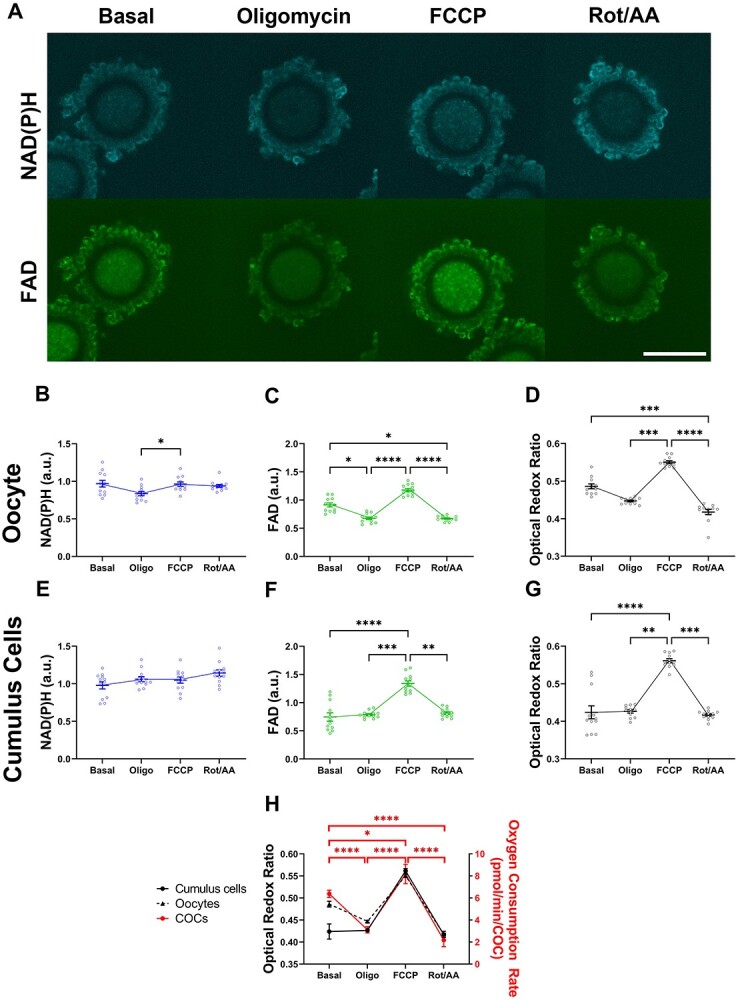
Optical imaging of autofluorescent metabolic cofactors in immature COCs reflects changes in oxygen consumption rate. Metabolism was measured in immature COCs in response to oligomycin (*oligo*: 2.0 μM), carbonyl cyanide-4-(trifluoromethoxy) phenylhydrazone (*FCCP*: 1 μM) and Rotenone/antimycin A (*Rot/AA*: 2.5 μM). The metabolic response to these inhibitors of the electron transport chain was measured by either laser scanning confocal microscopy (intracellular NAD(P)H and FAD) or extracellular flux analysis (oxygen consumption rate). Representative images of NAD(P)H and FAD autofluorescence are shown in (A). The intensity of NAD(P)H (B and E) and FAD (C and F) was quantified for the oocyte (B and C) and cumulus cells (E and F). The optical redox ratio (FAD / [NAD(P)H + FAD]) was calculated for the oocyte (D) and cumulus cells (G) as an indicator of overall metabolic activity. The optical redox ratio for the oocyte and cumulus cell compartments was compared with the oxygen consumption rate for whole COCs (H). Data are presented as mean ± SEM; optical redox ratio: *n* = 12 COCs per drug treatment, four independent experimental replicates; OCR: *n* = 16 wells (20 COCs/well; OCR was normalized using the number of COCs per well and presented as pmol/min/COC), four independent experimental replicates. Data were analyzed either by a Kruskal-Wallis with Dunn multiple comparison test (B and D) or a one-way ANOVA with Holm-Šídák multiple comparison test (C and E–G). ^*^P < 0.05, ^*^^*^P < 0.01, ^*^^*^^*^P < 0.001, ^*^^*^^*^^*^P < 0.0001. Scale bar = 80 μm.

We determined whether the optical redox ratio detects dynamic metabolic changes within immature COCs in response to mitochondrial inhibitors/uncoupler. We evaluated metabolic changes within the oocyte and cumulus cell compartments separately (Figure 1A). Compared to basal levels, there was a reduction in the intensity of NAD(P)H within the oocyte in response to oligomycin, although this did not reach statistical significance (Figure 1B). There was a significant reduction in FAD within the oocyte following the addition of oligomycin compared to basal levels (Figure 1C). The changes in NAD(P)H and FAD in response to oligomycin yielded no impact on the optical redox ratio of oocytes compared to basal levels (Figure 1D). Treatment with FCCP resulted in a significant increase in NAD(P)H, FAD, and optical redox ratio compared to levels observed in the presence of oligomycin (Figure 1B–D, respectively). Following addition of Rot/AA, there was no change in the levels of NAD(P)H but a significant decrease in FAD and optical redox ratio compared to levels seen in the presence of FCCP (Figure 1B–D, respectively).

**Figure 2:**
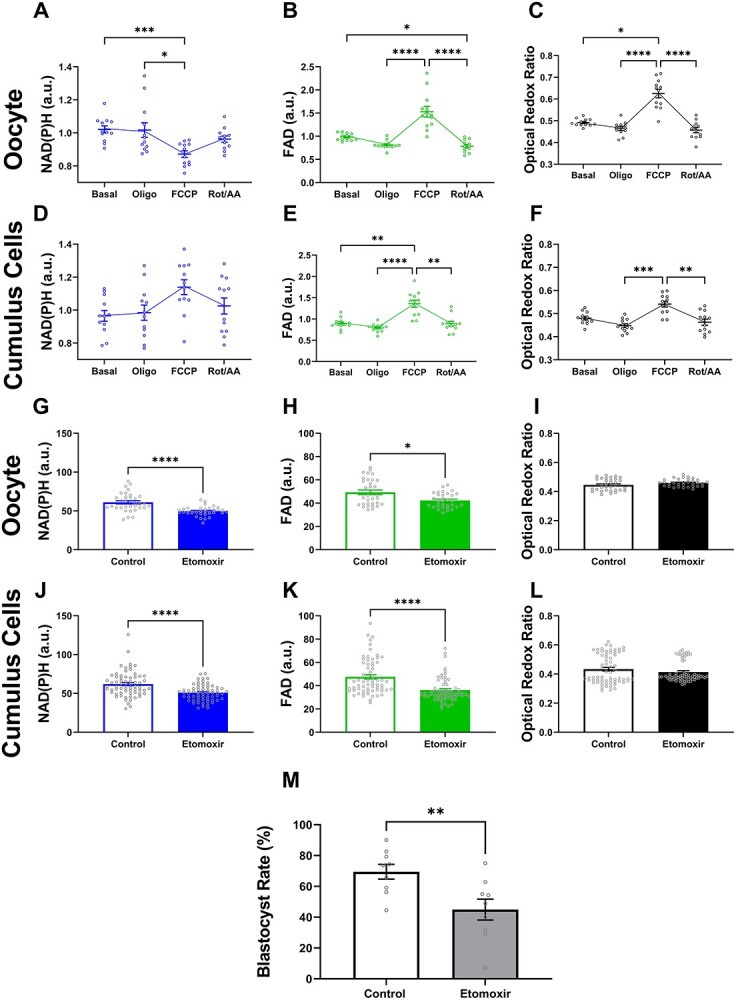
Optical imaging of metabolic cofactors in mature COCs is associated with oocyte quality. Metabolism was measured in mature COCs in response to oligomycin (*oligo*: 2.0 μM), carbonyl cyanide-4-(trifluoromethoxy) phenylhydrazone (*FCCP*: 1 μM) and Rotenone/antimycin A (*Rot/AA*: 2.5 μM). In a separate cohort, COCs were matured in vitro in the absence or presence of etomoxir, an inhibitor of fatty acid metabolism (β-oxidation). The metabolic response to inhibitors/uncoupler (A–F) or etomoxir (G–L) was measured by laser scanning confocal microscopy (intensity of NAD(P)H and FAD autofluorescence). The intensity of NAD(P)H (A, D, G, and J) and FAD (B, E, H, and K) was quantified in the oocyte (A, B, G, and H) and cumulus cells (D, E, J, and K). The optical redox ratio (FAD / [NAD(P)H + FAD]) was calculated as indicator of metabolic activity in oocytes (C and I) and cumulus cells (F and L). The effect of inhibiting fatty acid metabolism during IVM on oocyte developmental competence was assessed by subsequent development to the blastocyst stage (M; blastocyst-rate calculated from starting oocyte number). Data were analyzed by either a Kruskal-Wallis with Dunn multiple comparison test (*B*) or a one-way ANOVA with Holm-Šídák multiple comparison test (A and C–F) or two-tailed unpaired Student *t*-test (G and M) or Mann–Whitney test (H–L). Data presented as mean ± SEM, three independent experimental replicates; *n* = 12 COCs per treatment (A–F); *n* = 33 for control COCs, *n* = 32 for etomoxir-treated COCs (G–L); *n* = 174 and 138 embryos developed from control and etomoxir-treated COCs, respectively (M). ^*^P < 0.05, ^*^^*^P < 0.01, ^*^^*^^*^P < 0.001, ^*^^*^^*^^*^P < 0.0001.

In cumulus cells, the addition of oligomycin caused no changes in NAD(P)H, FAD, or the optical redox ratio compared to basal levels (Figure 1E–G, respectively). The addition of FCCP caused no change in the intensity of NAD(P)H but did result in a significant increase in FAD and the optical redox ratio compared to oligomycin (Figure 1E–G, respectively). Subsequent addition of Rot/AA yielded no change in the levels of NAD(P)H compared to FCCP but was significantly higher compared to basal levels (Figure 1E). For both FAD and the optical redox ratio, Rot/AA caused a significant decrease compared to FCCP levels (Figure 1F and G, respectively). Excitingly, when comparing the optical redox ratio with OCR, we observed that changes in optical redox ratio were consistent with OCR for the oocyte for all mitochondrial inhibitors/uncoupler (Figure 1H, *black dashed line* vs *red line*). The optical redox ratio for cumulus cells was consistent with changes in OCR with the exception of the response to oligomycin (Figure 1H; *black solid line* vs *red line*).

#### Optical imaging of metabolic cofactors in mature COCs detect metabolic changes associated with oocyte developmental competence

The above data demonstrated that label-free optical imaging of metabolic co-factors and subsequent calculation of the optical redox ratio were robust measures of metabolism in the immature oocyte. We next determined whether this approach was equivalently accurate for the mature COC (expanded, meiotically mature). In the oocyte, the addition of oligomycin yielded no impact on the intensity of NAD(P)H compared to basal levels (Figure 2A). Compared to basal levels, there was a decrease in FAD and optical redox ratio, although this was not statistically significant (Figure 2B and C, respectively). The addition of FCCP resulted in a significant decrease in the NAD(P)H and increase in the FAD compared to levels observed in the presence of oligomycin (Figure 2A and B, respectively). The changes in NAD(P)H and FAD in response to FCCP led to a significant increase in optical redox ratio compared to oligomycin (Figure 2C). Following addition of Rot/AA, there was no change in the intensity of NAD(P)H (Figure 2A) but there was a significant decrease of FAD and optical redox ratio compared to FCCP (Figure 2B and C, respectively).

In cumulus cells, there was no impact on NAD(P)H, FAD signal or optical redox ratio in response to oligomycin compared to basal levels (Figure 2D–F). The addition of FCCP caused no change in the level of NAD(P)H (Figure 2D) but resulted in a significant increase in FAD and the optical redox ratio compared to oligomycin (Figure 2E and F, respectively). Similar to FCCP, Rot/AA had no impact on the level of NAD(P)H (Figure 2D) but led to a significant decrease in the intensity of FAD and optical redox ratio (Figure 2E and F, respectively).

Toward determining whether this or similar optical approaches could detect metabolic changes associated with oocyte developmental potential, we utilized a well-described model of poor oocyte quality [9, 56]. COCs were matured in vitro in the absence or presence of etomoxir, which is known to inhibit fatty acid oxidation and result in decreased oocyte developmental potential [9]. The intensity of metabolic cofactors NAD(P)H and FAD was quantified in the oocyte and cumulus cells separately (Figure 2G–L). Compared to control, etomoxir treatment during IVM significantly reduced the intensity of NAD(P)H (Figure 2G and J) and FAD (Figure 2H and K) in both the oocyte (Figure 2G and H) and cumulus cells (Figure 2J and K). The presence of etomoxir during IVM did not alter the optical redox ratio for oocytes (Figure 2I) or cumulus cells (Figure 2L). Additionally, we confirmed that maturation in the presence of etomoxir negatively affects oocyte developmental potential. While maturation in the presence of etomoxir did not affect fertilization rate (control: 90.2 ± 2.5% vs etomoxir: 72.9 ± 8.8%, data not shown), it did result in significantly fewer embryos reaching the blastocyst stage of development (Figure 2M).

#### Hyperspectral microscopy detects metabolic changes associated with oocyte quality that persist in resultant blastocysts

**Figure 3:**
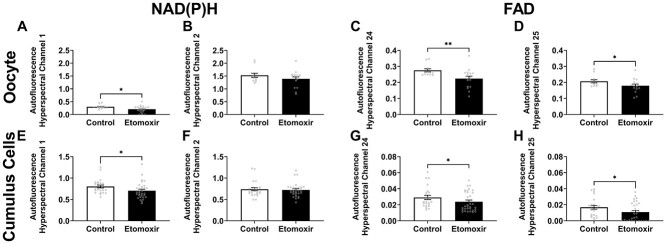
Hyperspectral microscopy detects metabolic changes associated with oocyte quality. COCs were matured in vitro in the absence or presence of etomoxir. Etomoxir is a known inhibitor of fatty acid metabolism (β-oxidation) and negatively affects oocyte developmental competence (see Figure 2M). Matured COCs were imaged using hyperspectral microscopy. Autofluorescence intensity was quantified for the oocyte (A–D) and cumulus cells (E–H) in hyperspectral channels that match the spectral properties of NAD(P)H: Channel 1 (A and E) and Channel 2 (B and F); and FAD: Channel 24 (C and G) and Channel 25 (D and H). Data were analyzed by a two-tailed unpaired Student *t*-test (A–C and H) or Mann–Whitney test (D–G). Data presented as mean ± SEM, three independent experimental replicates; *n* = 12 for control COCs, *n* = 16 for etomoxir-treated COCs. ^*^P < 0.05, ^*^^*^P < 0.01.

The use of laser-scanning confocal microscopy as a clinical measure of oocyte quality is hampered by the high laser energy dose required for imaging, likely resulting in photodamage [29]. Consequently, we next investigated whether hyperspectral microscopy could detect analogous changes in autofluorescence in the COC. Hyperspectral microscopy was chosen due to its 100-fold lower energy dose requirement compared to laser-scanning confocal imaging [35, 57]. Channels 1 and 2 were used to quantify the intensity of NAD(P)H, whereas Channels 24 and 25 were used to capture FAD (as described in *Materials and Methods*). Using the same model of poor oocyte quality, hyperspectral microscopy showed similar changes to NAD(P)H and FAD in both the oocyte and cumulus cells in response to etomoxir. Compared to control, there was a significant decrease in fluorescence in both the oocyte and cumulus cells following IVM in the presence of etomoxir in Channel 1 (Figure 3A and E, respectively) but no difference was seen in Channel 2 (Figure 3B and F, respectively). For both FAD channels (Channels 24 and 25) there was a significant decrease in autofluorescence in the oocyte and cumulus cells following IVM in the presence of etomoxir compared to control (oocyte: Figure 3C and D; cumulus cells: Figure 3G and H).

**Figure 4:**
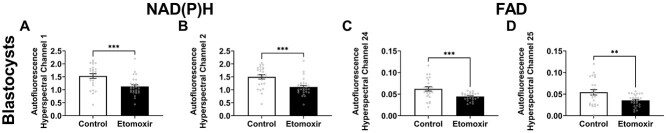
Altered metabolism during oocyte maturation persists in resultant blastocyst-stage embryos. COCs were matured in vitro in the absence or presence of etomoxir, an inhibitor of fatty acid metabolism (β-oxidation), which negatively affects oocyte developmental competence (see Figure 2M). Following in vitro maturation, COCs were fertilized in vitro and developed to the blastocyst stage in the absence of etomoxir. Embryos were imaged using hyperspectral microscopy. Autofluorescence intensity within the inner cell mass was quantified for hyperspectral channels that matched the spectral properties of NAD(P)H: Channel 1 (A) and Channel 2 (B); and FAD: Channel 24 (C) and Channel 25 (D). Data were analyzed by either a two-tailed unpaired Student *t*-test (C and D) or Mann–Whitney test (A and B). Data presented as mean ± SEM, three independent experimental replicates; *n* = 24 for control and *n* = 29 for blastocysts developed from control and etomoxir-treated COCs, respectively. ^*^^*^P < 0.01, ^*^^*^^*^P < 0.001.

To determine whether metabolic changes detected in etomoxir-treated oocytes persisted in resultant embryos, COCs matured in the absence or presence of etomoxir were fertilized in vitro and allowed to develop to the blastocyst stage. The intensity of NAD(P)H (Channels 1 and 2; Figure 4A and B) and FAD (Channels 24 and 25; Figure 4C and D) was significantly lower in the fetal cell lineage (inner cell mass) of blastocyst-stage embryos that developed from etomoxir-treated COCs compared to those matured in control conditions.

### Safety of hyperspectral imaging

**Figure 5:**
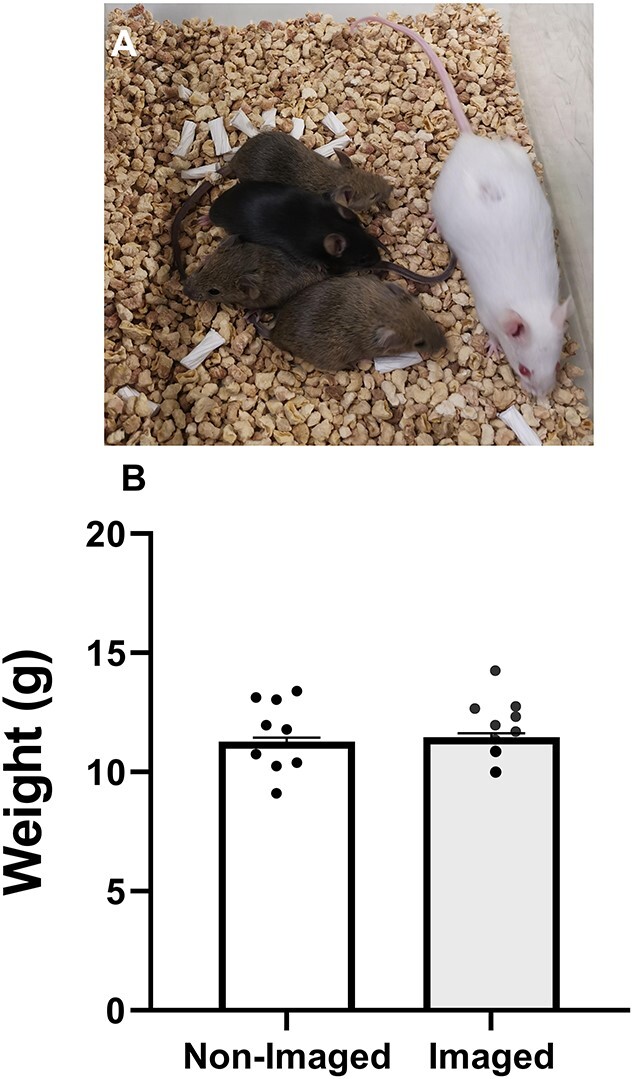
Hyperspectral imaging of the COC does not affect subsequent post-natal outcomes. Mature COCs were either imaged or not imaged using the hyperspectral microscope. COCs from both groups were fertilized in vitro and allowed to develop to the blastocyst-stage. Resultant blastocysts were transferred to pseudopregnant recipients with imaged COCs resulting in the birth of live pups (A). The weight of offspring at weaning was recorded for each group (B). Data are estimated marginal mean ± SEM and were analyzed by linear mixed model with litter size as a covariate. Each datum point represents the average weight per litter. *n* = 9 L per group. See Supplementary Figure SS3 for offspring data according to sex.

To assess whether photodamage had occurred in response to imaging, untreated mature COCs were either not imaged (*non-imaged*) or imaged using hyperspectral autofluorescence microscopy (*imaged*). Both groups of COCs were then fertilized in vitro and allowed to develop to the blastocyst-stage. Imaging of COCs did not affect fertilization rate (non-imaged: 90.15 ± 1.71% vs imaged: 84.49 ± 2.76%, data not shown) or subsequent development to the blastocyst stage (non-imaged: 63.47 ± 5.18% vs imaged: 61.87 ± 4.78%, data not shown). We next examined whether hyperspectral imaging of the COC altered the potential of the subsequent embryo to implant and result in the birth of a healthy live off-spring. Imaged COCs resulted in the birth of live pups (Figure 5A). There were no significant differences in pregnancy or live birth rate (*P* > 0.05; Supplementary Table SS2). Imaged versus non-imaged COCs resulted in offspring with similar, and not significantly different, weights at weaning (Figure 5B; Supplementary Table SS3). Similarly, there was no difference in weight at weaning according to sex (Supplementary Figure SS3). No gross facial deformities were noted across treatment groups.

## Discussion

Most metabolism assays are limited in that they assess the whole COC and fail to provide spatial information on the oocyte and cumulus cell compartments [2]. Development of a tool that non-invasively measures metabolism in both compartments may be a powerful route for assessing oocyte quality—particularly as it would provide a measurement of the oocyte compartment of the COC. Label-free optical imaging has been previously used to characterize good and poor quality oocytes that were denuded of their cumulus cells [33, 36, 56, 58]. However, as oocytes are dependent on factors derived from cumulus cells and normally mature in the presence of these cells [2], it is important to validate the robustness of label-free optical imaging to measure metabolism in the intact COC. This was analyzed in this study by comparing the optical redox ratio of the cumulus cells and oocyte with the rate of oxygen consumption in the whole COC, and in response to a series of mitochondrial inhibitors/uncoupler. Following this, we showed that optical imaging using confocal microscopy could detect metabolic differences associated with oocyte developmental potential. Toward demonstrating the potential for label-free optical imaging to be used clinically, we also used hyperspectral microscopy, which typically uses light at 1–2 orders of magnitude lower than confocal microscopy [35, 57]. This makes it compatible for clinical use due to the absence of photodamage as shown in this and our previous study [49]. Importantly, results generated with hyperspectral imaging were comparable to those obtained from confocal microscopy.

Our results showed that the optical redox ratio is an accurate assay to measure metabolic changes in the oocyte through our comparison with oxygen consumption. In cumulus cells, the response to the inhibitors/uncoupler yielded similar changes in optical redox ratio and oxygen consumption, with the exception of oligomycin. Oligomycin inhibits ATP synthase in the electron transport chain (Supplementary Figure SS3), leading to a decrease in oxygen consumption at complex IV, which was detected by the extracellular flux assay. Conversely, in cumulus cells the optical redox ratio did not change in response to oligomycin. This may be due to oligomycin causing a shift in metabolism from oxidative phosphorylation to glycolysis [59]. An increase in glycolysis would lead to elevated levels of NADH in the cytosol and a resultant increase in NAD(P)H fluorescence. A shift to glycolysis would not occur within the oocyte as it lacks active phosphofructokinase: the rate limiting step in glycolysis [2, 33, 60]. Future studies could utilize metabolic inhibitors such as 2-deoxy-d-glucose and oxamate to understand the impact of glycolysis on the levels of NAD(P)H and FAD in the cumulus cells.

It is important to note that the signal intensity of NAD(P)H captured here may potentially be a mixture of both NADH and NADPH due to their near identical spectral properties [29, 31]. Therefore, the results observed for NAD(P)H could potentially be attributed to (1) NADH produced from glycolysis and the tricaboxylic (TCA) cycle to generate ATP in the electron transport chain [29], or (2) cytosolic NADPH from the pentose phosphate pathway in response to oxidative stress [61, 62]. In contrast, FAD can be directly linked to the activity of oxidative phosphorylation, as it is almost exclusively localized within mitochondria [29, 63]. Importantly, we showed that changes in FAD intensity in response to the mitochondrial inhibitors/uncoupler were as hypothesized for the oocyte and cumulus cells—except for oligomycin in cumulus cells as discussed above. Thus, recording FAD autofluorescence is an accurate measurement of oxidative phosphorylation, particularly for the oocyte. This demonstrates the power of label-free optical imaging to interrogate differences in metabolism between the oocyte and cumulus cells, while the OCR is limited in that it measures metabolism of the entire COC, potentially missing critical differences between the two cell compartments.

The capacity of label-free optical imaging to detect metabolic differences associated with oocyte quality was demonstrated in this study using a mouse model of poor oocyte quality—etomoxir-induced inhibition of fatty acid oxidation during IVM. The importance of fatty acid oxidation for oocyte developmental potential was shown in this and previous studies [9, 10, 64]. While etomoxir does not directly target oxidative phosphorylation, we hypothesized that it would lead to a decrease in NAD(P)H and FAD as fatty acids are normally metabolized to acetyl coenzyme A to generate NADH and FADH_2_ in the TCA cycle [9]. These metabolic cofactors can then be used in oxidative phosphorylation to generate ATP (Supplementary Figure SS4). As expected, we observed a decrease in NAD(P)H and FAD intensities when COCs were matured in the presence of etomoxir. This demonstrates the premise of label-free imaging to detect metabolic differences in COCs associated with oocyte quality. We are now turning our attention to a wide range of models, both animal and human, where oocyte competence is known to be an issue.

To demonstrate clinical utility, we also used hyperspectral microscopy that requires low energy dose for imaging. In addition to confirming the results obtained from confocal imaging in the COC, hyperspectral microscopy was able to detect altered metabolism in blastocysts developed from oocytes with poor developmental potential. This shows that insults that occur during IVM persist in resultant blastocysts as seen in previous work when the insult occurred during oocyte maturation in vivo [65].

Importantly, we assessed the safety of imaging the COC using hyperspectral microscopy. This is of critical importance as previous studies have shown that light exposure can be harmful for the developing embryo [66–68]. Our results showed that exposure of COCs to this imaging modality had no impact on the ability of subsequent embryos to develop to the blastocyst stage, implant, or result in the birth of a live, healthy offspring. This is comparable to our results seen following hyperspectral imaging of the preimplantation embryo [49] and reaffirms the clinical potential of this imaging modality.

Several imaging modalities are used to record autofluorescence from intracellular FAD and NAD(P)H including laser scanning confocal microscopy, fluorescence lifetime imaging, and hyperspectral microscopy [33–35, 49, 54, 69, 70]. The use of single channel optical imaging in this study is an accepted means of recording autofluorescence from these metabolic co-factors both in our field [33, 34, 36] and others [30, 32, 71, 72]. Furthermore, this study also validates the use of this approach. In response to the mitochondrial inhibitors, changes in the optical redox ratio ($\mathrm{FAD}/\ [\mathrm{NAD}(\mathrm{P})\mathrm{H}+\mathrm{FAD}])$were as hypothesized for the oocyte. This was also true for cumulus cells with the exception of oligomycin, the reasons for which are discussed above.

It is important to note that this study was performed in a mouse model. Further evaluation of this imaging tool in preclinical studies and additional safety assessments in larger animal species are required prior to clinical implementation. As the developmental potential of an embryo is heavily reliant on the oocyte it is derived from, non-invasive selection and ranking of oocytes may assist in optimizing an IVF cycle to increase the likelihood of success [73]. This study demonstrates that label-free optical imaging of NAD(P)H and FAD is a sensitive assay for measuring metabolism in the oocyte and detects metabolic signatures associated with oocyte quality. We believe that label-free optical imaging is a promising technique for measuring oocyte developmental potential, and potentially improving IVF success.

## Data availability

The data underlying this article will be shared on reasonable request to the corresponding author.

## Authorship contributions

H.M.B., J.G.T., and K.R.D. conceived the idea for the study. T.C.Y.T., S.M., and K.R.D. were involved in the experimental design. T.C.Y.T. was involved in data acquisition, generation of figures, and data analysis. T.C.Y.T., S.M., and K.R.D. were involved in the interpretation of data. T.C.Y.T. and K.R.D. wrote the first draft and most of the manuscript. All authors critically reviewed and edited the manuscript and approved the final version.

## Supplementary Material

Supp_fig_Assessment_of_oocyte_quality_ioac145

Supp_table_Assessment_of_oocyte_quality_ioac145

## References

[1] Conti M, Franciosi F. Acquisition of oocyte competence to develop as an embryo: integrated nuclear and cytoplasmic events. Hum Reprod Update 2018; 24:245–266. 29432538 10.1093/humupd/dmx040 PMC5907346

[2] Richani D, Dunning KR, Thompson JG, Gilchrist RB. Metabolic co-dependence of the oocyte and cumulus cells: essential role in determining oocyte developmental competence. Hum Reprod Update 2021; 27:27–47. 33020823 10.1093/humupd/dmaa043

[3] Coticchio G, Sereni E, Serrao L, Mazzone S, Iadarola I, Borini A. What criteria for the definition of oocyte quality? Ann N Y Acad Sci 2004; 1034:132–144. 15731306 10.1196/annals.1335.016

[4] Wang Q, Sun QY. Evaluation of oocyte quality: morphological, cellular and molecular predictors. Reprod Fertil Dev 2007; 19:1–12. 10.1071/rd06103 17389130

[5] Wong KM, Repping S, Mastenbroek S. Limitations of embryo selection methods. Semin Reprod Med 2014; 32:127–133. 24515907 10.1055/s-0033-1363554

[6] O'Brien JK, Dwarte D, Ryan JP, Maxwell WM, Evans G. Developmental capacity, energy metabolism and ultrastructure of mature oocytes from prepubertal and adult sheep. Reprod Fertil Dev 1996; 8:1029–1037. 8916278 10.1071/rd9961029

[7] Sutton-McDowall ML, Gilchrist RB, Thompson JG. The pivotal role of glucose metabolism in determining oocyte developmental competence. Reproduction 2010; 139:685–695. 20089664 10.1530/REP-09-0345

[8] Hemmings KE, Leese HJ, Picton HM. Amino acid turnover by bovine oocytes provides an index of oocyte developmental competence in vitro. Biol Reprod 2012; 86(5):165, 1–12. 10.1095/biolreprod.111.092585 22378762

[9] Dunning KR, Cashman K, Russell DL, Thompson JG, Norman RJ, Robker RL. Beta-oxidation is essential for mouse oocyte developmental competence and early embryo development. Biol Reprod 2010; 83:909–918. 20686180 10.1095/biolreprod.110.084145

[10] Ferguson EM, Leese HJ. A potential role for triglyceride as an energy source during bovine oocyte maturation and early embryo development. Mol Reprod Dev 2006; 73:1195–1201. 16804881 10.1002/mrd.20494

[11] Wu LL, Dunning KR, Yang X, Russell DL, Lane M, Norman RJ, Robker RL. High-fat diet causes lipotoxicity responses in cumulus-oocyte complexes and decreased fertilization rates. Endocrinology 2010; 151:5438–5445. 20861227 10.1210/en.2010-0551

[12] Van Blerkom J, Davis PW, Lee J. ATP content of human oocytes and developmental potential and outcome after in-vitro fertilization and embryo transfer. Hum Reprod 1995; 10:415–424. 7769073 10.1093/oxfordjournals.humrep.a135954

[13] Stojkovic M, Machado SA, Stojkovic P, Zakhartchenko V, Hutzler P, Goncalves PB, Wolf E. Mitochondrial distribution and adenosine triphosphate content of bovine oocytes before and after in vitro maturation: correlation with morphological criteria and developmental capacity after in vitro fertilization and culture. Biol Reprod 2001; 64:904–909. 11207207 10.1095/biolreprod64.3.904

[14] Nagano M, Katagiri S, Takahashi Y. ATP content and maturational/developmental ability of bovine oocytes with various cytoplasmic morphologies. Zygote 2006; 14:299–304. 17266788 10.1017/S0967199406003807

[15] Dalton CM, Szabadkai G, Carroll J. Measurement of ATP in single oocytes: impact of maturation and cumulus cells on levels and consumption. J Cell Physiol 2014; 229:353–361. 24002908 10.1002/jcp.24457

[16] Robker RL, Akison LK, Bennett BD, Thrupp PN, Chura LR, Russell DL, Lane M, Norman RJ. Obese women exhibit differences in ovarian metabolites, hormones, and gene expression compared with moderate-weight women. J Clin Endocrinol Metab 2009; 94:1533–1540. 19223519 10.1210/jc.2008-2648

[17] Fragouli E, Wells D, Iager AE, Kayisli UA, Patrizio P. Alteration of gene expression in human cumulus cells as a potential indicator of oocyte aneuploidy. Hum Reprod 2012; 27:2559–2568. 22617123 10.1093/humrep/des170

[18] Gebhardt KM, Feil DK, Dunning KR, Lane M, Russell DL. Human cumulus cell gene expression as a biomarker of pregnancy outcome after single embryo transfer. Fertil Steril 2011; 96:e42. 21575950 10.1016/j.fertnstert.2011.04.033

[19] Bracewell-Milnes T, Saso S, Abdalla H, Nikolau D, Norman-Taylor J, Johnson M, Holmes E, Thum M-Y. Metabolomics as a tool to identify biomarkers to predict and improve outcomes in reproductive medicine: a systematic review. Hum Reprod Update 2017; 23:723–736. 29069503 10.1093/humupd/dmx023

[20] Walls ML, Hunter T, Ryan JP, Keelan JA, Nathan E, Hart RJ. In vitro maturation as an alternative to standard in vitro fertilization for patients diagnosed with polycystic ovaries: a comparative analysis of fresh, frozen and cumulative cycle outcomes. Hum Reprod 2015; 30:88–96. 25355587 10.1093/humrep/deu248

[21] Harris SE, Leese HJ, Gosden RG, Picton HM. Pyruvate and oxygen consumption throughout the growth and development of murine oocytes. Mol Reprod Dev 2009; 76:231–238. 18618608 10.1002/mrd.20945

[22] Tejera A, Herrero J, Santos M, Garrido N, Ramsing N, Meseguer M. Oxygen consumption is a quality marker for human oocyte competence conditioned by ovarian stimulation regimens. Fertil Steril 2011; 96:618–623.e612. 21782167 10.1016/j.fertnstert.2011.06.059

[23] Brand MD, Nicholls DG. Assessing mitochondrial dysfunction in cells. Biochem J 2011; 435:297–312. 21726199 10.1042/BJ20110162 PMC3076726

[24] Houghton FD, Thompson JG, Kennedy CJ, Leese HJ. Oxygen consumption and energy metabolism of the early mouse embryo. Mol Reprod Dev 1996; 44:476–485. 8844690 10.1002/(SICI)1098-2795(199608)44:4<476::AID-MRD7>3.0.CO;2-I

[25] Obeidat YM, Evans AJ, Tedjo W, Chicco AJ, Carnevale E, Chen TW. Monitoring oocyte/embryo respiration using electrochemical-based oxygen sensors. Sens Actuators B 2018; 276:72–81.

[26] Thompson JG, Partridge RJ, Houghton FD, Cox CI, Leese HJ. Oxygen uptake and carbohydrate metabolism by in vitro derived bovine embryos. J Reprod Fertil 1996; 106:299–306. 8699414 10.1530/jrf.0.1060299

[27] Croce AC, Bottiroli G. Autofluorescence spectroscopy and imaging: a tool for biomedical research and diagnosis. Eur J Histochem 2014; 58:2461. 25578980 10.4081/ejh.2014.2461 PMC4289852

[28] Kolenc OI, Quinn KP. Evaluating cell metabolism through autofluorescence imaging of NAD(P)H and FAD. Antioxid Redox Signal 2019; 30:875–889. 29268621 10.1089/ars.2017.7451 PMC6352511

[29] Georgakoudi I, Quinn KP. Optical imaging using endogenous contrast to assess metabolic state. Annu Rev Biomed Eng 2012; 14:351–367. 22607264 10.1146/annurev-bioeng-071811-150108

[30] Skala MC, Riching KM, Gendron-Fitzpatrick A, Eickhoff J, Eliceiri KW, White JG, Ramanujam N. In vivo multiphoton microscopy of NADH and FAD redox states, fluorescence lifetimes, and cellular morphology in precancerous epithelia. Proc Natl Acad Sci U S A 2007; 104:19494–19499. 18042710 10.1073/pnas.0708425104 PMC2148317

[31] Galeotti T, van Rossum GD, Mayer DH, Chance B. On the fluorescence of NAD(P)H in whole-cell preparations of tumours and normal tissues. Eur J Biochem 1970; 17:485–496. 4395441 10.1111/j.1432-1033.1970.tb01191.x

[32] Chance B, Schoener B, Oshino R, Itshak F, Nakase Y. Oxidation-reduction ratio studies of mitochondria in freeze-trapped samples. NADH and flavoprotein fluorescence signals. J Biol Chem 1979; 254:4764–4771. 220260

[33] Dumollard R, Marangos P, Fitzharris G, Swann K, Duchen M, Carroll J. Sperm-triggered [Ca2+] oscillations and Ca2+ homeostasis in the mouse egg have an absolute requirement for mitochondrial ATP production. Development 2004; 131:3057–3067. 15163630 10.1242/dev.01181

[34] Dumollard R, Ward Z, Carroll J, Duchen MR. Regulation of redox metabolism in the mouse oocyte and embryo. Development 2007; 134:455–465. 17185319 10.1242/dev.02744

[35] Sutton-McDowall ML, Gosnell M, Anwer AG, White M, Purdey M, Abell AD, Goldys EM, Thompson JG. Hyperspectral microscopy can detect metabolic heterogeneity within bovine post-compaction embryos incubated under two oxygen concentrations (7% versus 20%). Hum Reprod 2017; 32:2016–2025. 28938734 10.1093/humrep/dex261

[36] Sutton-McDowall ML, Wu LL, Purdey M, Abell AD, Goldys EM, MacMillan KL, Thompson JG, Robker RL. Nonesterified fatty acid-induced endoplasmic reticulum stress in cattle cumulus oocyte complexes alters cell metabolism and developmental competence. Biol Reprod 2016; 94:23. 26658709 10.1095/biolreprod.115.131862

[37] Pike Winer LS, Wu M. Rapid analysis of glycolytic and oxidative substrate flux of cancer cells in a microplate. PLoS One 2014; 9:e109916. 25360519 10.1371/journal.pone.0109916 PMC4215881

[38] Bhatia S, Thompson EW, Gunter JH. Studying the metabolism of epithelial-mesenchymal plasticity using the seahorse XFe96 extracellular flux analyzer. Methods Mol Biol 2021; 2179:327–340. 32939731 10.1007/978-1-0716-0779-4_25

[39] Martin SD, McGee SL. A systematic flux analysis approach to identify metabolic vulnerabilities in human breast cancer cell lines. Cancer Metab 2019; 7:12. 31890204 10.1186/s40170-019-0207-x PMC6935091

[40] Li CJ, Lin LT, Tsui KH. Dehydroepiandrosterone shifts energy metabolism to increase mitochondrial biogenesis in female fertility with advancing age. Nutrients 2021; 13(7):2449. 10.3390/nu13072449 PMC8308577 34371958

[41] Muller B, Lewis N, Adeniyi T, Leese HJ, Brison DR, Sturmey RG. Application of extracellular flux analysis for determining mitochondrial function in mammalian oocytes and early embryos. Sci Rep 2019; 9:16778. 31727902 10.1038/s41598-019-53066-9 PMC6856134

[42] Balbach M, Gervasi MG, Hidalgo DM, Visconti PE, Levin LR, Buck J. Metabolic changes in mouse sperm during capacitationdagger. Biol Reprod 2020; 103:791–801. 32614044 10.1093/biolre/ioaa114 PMC7822642

[43] Soto-Heras S, Menendez-Blanco I, Catala MG, Izquierdo D, Thompson JG, Paramio MT. Biphasic in vitro maturation with C-type natriuretic peptide enhances the developmental competence of juvenile-goat oocytes. PLoS One 2019; 14:e0221663. 31442286 10.1371/journal.pone.0221663 PMC6707569

[44] Zhang M, Su YQ, Sugiura K, Wigglesworth K, Xia G, Eppig JJ. Estradiol promotes and maintains cumulus cell expression of natriuretic peptide receptor 2 (NPR2) and meiotic arrest in mouse oocytes in vitro. Endocrinology 2011; 152:4377–4385. 21914782 10.1210/en.2011-1118 PMC3199003

[45] Zeng H-t, Ren Z, Guzman L, Wang X, Sutton-McDowall ML, Ritter LJ, De Vos M, Smitz J, Thompson JG, Gilchrist RB. Heparin and cAMP modulators interact during pre-in vitro maturation to affect mouse and human oocyte meiosis and developmental competence. Hum Reprod 2013; 28:1536–1545. 23559189 10.1093/humrep/det086

[46] Lim M, Brown HM, Rose RD, Thompson JG, Dunning KR. Dysregulation of bisphosphoglycerate mutase during in vitro maturation of oocytes. J Assist Reprod Genet 2021; 38:1363–1372. 34052998 10.1007/s10815-021-02230-0 PMC8266955

[47] Dunning KR, Lane M, Brown HM, Yeo C, Robker RL, Russell DL. Altered composition of the cumulus-oocyte complex matrix during in vitro maturation of oocytes. Hum Reprod 2007; 22:2842–2850. 17872911 10.1093/humrep/dem277

[48] Lim M, Brown HM, Kind KL, Breen J, Anastasi MR, Ritter LJ, Tregoweth EK, Dinh DT, Thompson JG, Dunning KR. Haemoglobin expression in in vivo murine preimplantation embryos suggests a role in oxygen-regulated gene expression. Reprod Fertil Dev 2019; 31:724–734. 30482269 10.1071/RD17321

[49] Tan TCY, Mahbub SB, Campbell JM, Habibalahi A, Campugan CA, Rose RD, Chow DJX, Mustafa S, Goldys EM, Dunning KR. Non-invasive, label-free optical analysis to detect aneuploidy within the inner cell mass of the preimplantation embryo. Hum Reprod 2022; 37:14–29. 10.1093/humrep/deab233 34741175

[50] Mahbub SB, Tomczyk J, Goldys EM. GUI_Preprocess v3.12 2020.

[51] Habibalahi A, Bala C, Allende A, Anwer AG, Goldys EM. Novel automated non invasive detection of ocular surface squamous neoplasia using multispectral autofluorescence imaging. Ocul Surf 2019; 17:540–550. 30904597 10.1016/j.jtos.2019.03.003

[52] Mahbub SB, Ploschner M, Gosnell ME, Anwer AG, Goldys EM. Statistically strong label-free quantitative identification of native fluorophores in a biological sample. Sci Rep 2017; 7:15792. 29150629 10.1038/s41598-017-15952-y PMC5693869

[53] Rehman AU, Anwer AG, Gosnell ME, Mahbub SB, Liu G, Goldys EM. Fluorescence quenching of free and bound NADH in HeLa cells determined by hyperspectral imaging and unmixing of cell autofluorescence. Biomed Opt Express 2017; 8:1488. 28663844 10.1364/BOE.8.001488 PMC5480559

[54] Blacker TS, Mann ZF, Gale JE, Ziegler M, Bain AJ, Szabadkai G, Duchen MR. Separating NADH and NADPH fluorescence in live cells and tissues using FLIM. Nat Commun 2014; 5:3936. 24874098 10.1038/ncomms4936 PMC4046109

[55] Salabei JK, Gibb AA, Hill BG. Comprehensive measurement of respiratory activity in permeabilized cells using extracellular flux analysis. Nat Protoc 2014; 9:421–438. 24457333 10.1038/nprot.2014.018 PMC4063296

[56] Bradley J, Pope I, Wang Y, Langbein W, Borri P, Swann K. Dynamic label-free imaging of lipid droplets and their link to fatty acid and pyruvate oxidation in mouse eggs. J Cell Sci 2019; 132(13):jcs228999. 10.1242/jcs.228999 31182643

[57] Jonkman J, Brown CM. Any way you slice it-A comparison of confocal microscopy techniques. J Biomol Tech 2015; 26:54–65. 25802490 10.7171/jbt.15-2602-003 PMC4365987

[58] Zeng HT, Richani D, Sutton-McDowall ML, Ren Z, Smitz JE, Stokes Y, Gilchrist RB, Thompson JG. Prematuration with cyclic adenosine monophosphate modulators alters cumulus cell and oocyte metabolism and enhances developmental competence of in vitro-matured mouse oocytes. Biol Reprod 2014; 91:47. 24966394 10.1095/biolreprod.114.118471

[59] Connolly NMC, Theurey P, Adam-Vizi V, Bazan NG, Bernardi P, Bolanos JP, Culmsee C, Dawson VL, Deshmukh M, Duchen MR, Dussmann H, Fiskum G et al. Guidelines on experimental methods to assess mitochondrial dysfunction in cellular models of neurodegenerative diseases. Cell Death Differ 2018; 25:542–572. 29229998 10.1038/s41418-017-0020-4 PMC5864235

[60] Biggers JD, Whittingham DG, Donahue RP. The pattern of energy metabolism in the mouse oocyte and zygote. Proc Natl Acad Sci U S A 1967; 58:560–567. 5233459 10.1073/pnas.58.2.560 PMC335672

[61] Ying W . NAD+/NADH and NADP+/NADPH in cellular functions and cell death: regulation and biological consequences. Antioxid Redox Signal 2008; 10:179–206. 18020963 10.1089/ars.2007.1672

[62] May-Panloup P, Boguenet M, Hachem HE, Bouet PE, Reynier P. Embryo and its mitochondria. Antioxidants (Basel) 2021; 10(2):139. 10.3390/antiox10020139 PMC7908991 33498182

[63] Kunz WS, Kunz W. Contribution of different enzymes to flavoprotein fluorescence of isolated rat liver mitochondria. Biochim Biophys Acta 1985; 841:237–246. 4027266 10.1016/0304-4165(85)90064-9

[64] Sturmey RG, O'Toole PJ, Leese HJ. Fluorescence resonance energy transfer analysis of mitochondrial:lipid association in the porcine oocyte. Reproduction 2006; 132:829–837. 17127743 10.1530/REP-06-0073

[65] Minge CE, Bennett BD, Norman RJ, Robker RL. Peroxisome proliferator-activated receptor-gamma agonist rosiglitazone reverses the adverse effects of diet-induced obesity on oocyte quality. Endocrinology 2008; 149:2646–2656. 18276752 10.1210/en.2007-1570

[66] Bognar Z, Csabai TJ, Pallinger E, Balassa T, Farkas N, Schmidt J, Gorgey E, Berta G, Szekeres-Bartho J, Bodis J. The effect of light exposure on the cleavage rate and implantation capacity of preimplantation murine embryos. J Reprod Immunol 2019; 132:21–28. 30852462 10.1016/j.jri.2019.02.003

[67] Khodavirdilou R, Pournaghi M, Oghbaei H, Rastgar Rezaei Y, Javid F, Khodavirdilou L, Shakibfar F, Latifi Z, Hakimi P, Nouri M, Fattahi A, Dittrich R. Toxic effect of light on oocyte and pre-implantation embryo: a systematic review. Arch Toxicol 2021; 95:3161–3169. 34448882 10.1007/s00204-021-03139-4

[68] Takenaka M, Horiuchi T, Yanagimachi R. Effects of light on development of mammalian zygotes. Proc Natl Acad Sci U S A 2007; 104:14289–14293. 17709739 10.1073/pnas.0706687104 PMC1964859

[69] Santos Monteiro CA, Chow DJX, Leal GR, Tan TC, Reis Ferreira AM, Thompson JG, Dunning KR. Optical imaging of cleavage stage bovine embryos using hyperspectral and confocal approaches reveals metabolic differences between on-time and fast-developing embryos. Theriogenology 2021; 159:60–68. 33113445 10.1016/j.theriogenology.2020.10.012

[70] Sanchez T, Venturas M, Aghvami SA, Yang X, Fraden S, Sakkas D, Needleman DJ. Combined noninvasive metabolic and spindle imaging as potential tools for embryo and oocyte assessment. Hum Reprod 2019; 34:2349–2361. 31812992 10.1093/humrep/dez210 PMC6936724

[71] Skala M, Ramanujam N. Multiphoton redox ratio imaging for metabolic monitoring in vivo. Methods Mol Biol 2010; 594:155–162. 20072916 10.1007/978-1-60761-411-1_11 PMC2874879

[72] Ostrander JH, McMahon CM, Lem S, Millon SR, Brown JQ, Seewaldt VL, Ramanujam N. Optical redox ratio differentiates breast cancer cell lines based on estrogen receptor status. Cancer Res 2010; 70:4759–4766. 20460512 10.1158/0008-5472.CAN-09-2572 PMC3826951

[73] Chow DJX, Wijesinghe P, Dholakia K, Dunning KR. Does artificial intelligence have a role in the IVF clinic? Reprod Fertil 2021; 2:C29–C34. 35118395 10.1530/RAF-21-0043 PMC8801019

