# Supplementary material for: Optical imaging detects metabolic signatures associated with oocyte quality
Source: Biol Reprod. 2022 Jul 21;107(4):1014–25. doi: 10.1093/biolre/ioac145 (PMC9562116; doi:10.1093/biolre/ioac145)
Supplement: Supp_fig_Assessment_of_oocyte_quality_ioac145 [file supp_fig_assessment_of_oocyte_quality_ioac145.docx]

# Supplementary Figures

**
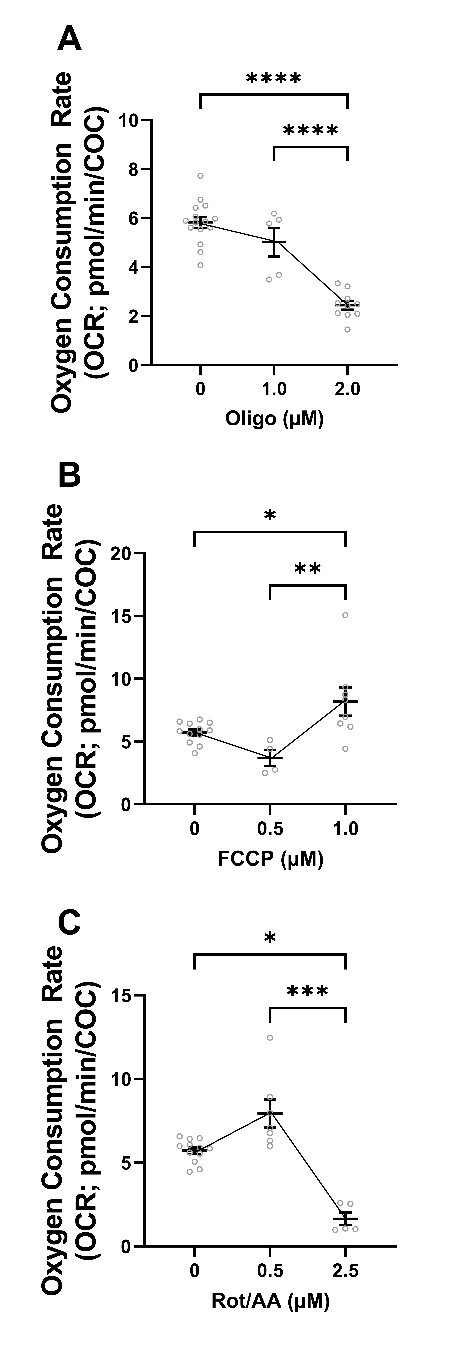
**

**Supplementary Figure 1. Optimization of inhibitor/uncoupler concentration to examine the contribution of specific components of the electron transport chain on oxygen consumption in cumulus oocyte complexes.** Oxygen consumption rate (OCR) was measured in immature cumulus oocyte complexes (COCs) in response to different concentrations of oligomycin (*oligo*: 0, 1.0 μM or 2.0 μM); carbonyl cyanide-4-(trifluoromethoxy) phenylhydrazone (*FCCP*: 0, 0.5 μM or 1.0 μM) and Rotenone/antimycin A (*Rot/AA*: 0, 0.5 μM or 2.5 μM). Data presented as mean ± SEM. Data were analyzed by a Kruskal-Wallis with Dunn’s multiple comparison test (**C**) or a one – way ANOVA with Holm-Šídák multiple comparison test (**A** and **B**). n = 5-15 wells for each concentration (20 COCs /well; oxygen consumption rate was normalized using the number of COCs per well and presented as pmol/min/COC). * *P* < 0.05, ** *P* < 0.01, *** *P* < 0.001, **** *P* < 0.0001.


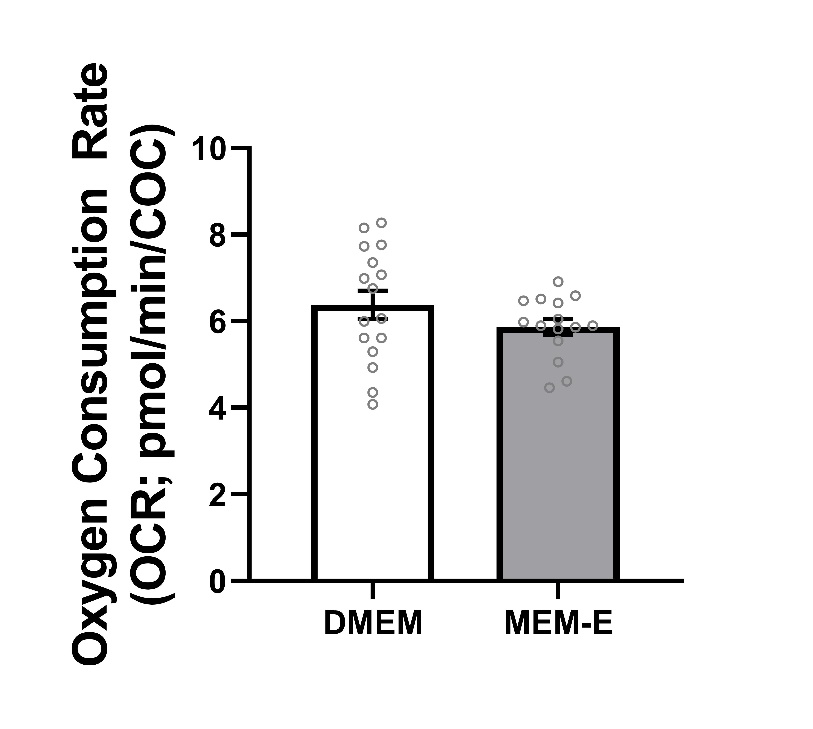


**Supplementary Figure 2. Oxygen consumption rate in cumulus oocyte complexes cultured in Seahorse XF DMEM medium or Minimum Essential Medium-Eagle.** Oxygen consumption rate (OCR) was measured in immature cumulus oocyte complexes (COCs) in Seahorse XF DMEM or Minimum Essential Medium-Eagle (MEM-E). Data presented as mean ± SEM. Data were analyzed by a two-tailed unpaired Student’s t-test. n = 16 wells for DMEM and 15 wells for MEM-E (20 COCs /well; oxygen consumption rate was normalized using the number of COCs per well and presented as pmol/min/COC).


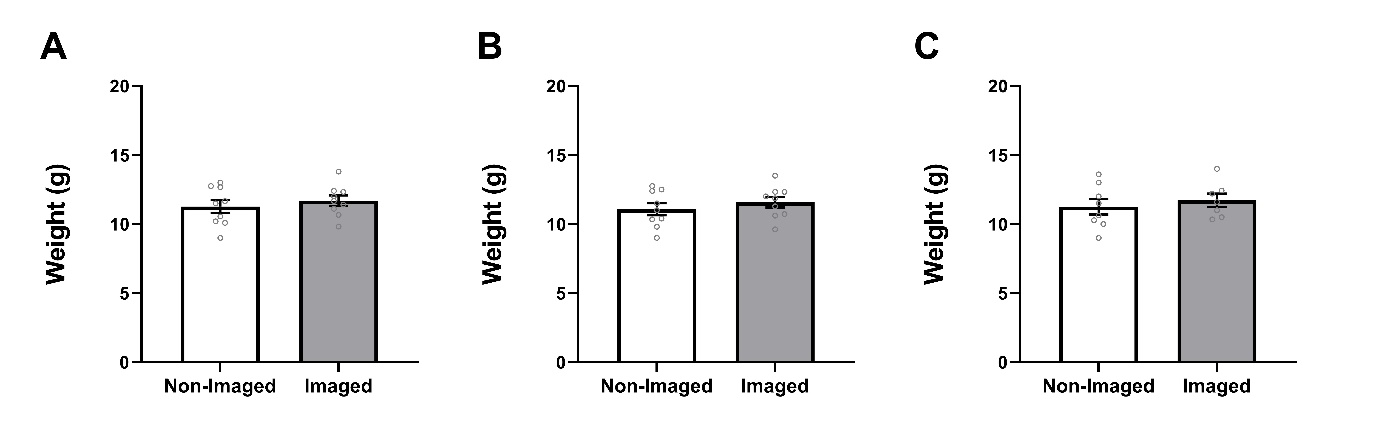


**Supplementary Figure 3. Hyperspectral imaging of the cumulus oocyte complex does not affect weight at weaning according to sex.** Mature cumulus oocyte complexes (COCs) were either imaged or not imaged using the hyperspectral microscope. COCs from both groups were fertilized in vitro and allowed to develop to the blastocyst-stage. Resultant blastocysts were transferred to pseudopregnant recipients. The weight of offspring at weaning were recorded for each group. Each datum point represents the average weight per litter (A) or average weight of female offspring per litter (B) or average weight of male offspring per litter (C). Data are presented as mean ± SEM. Data were analyzed by linear mixed model with litter size as a covariate. *n* = 7-9 litter per group.


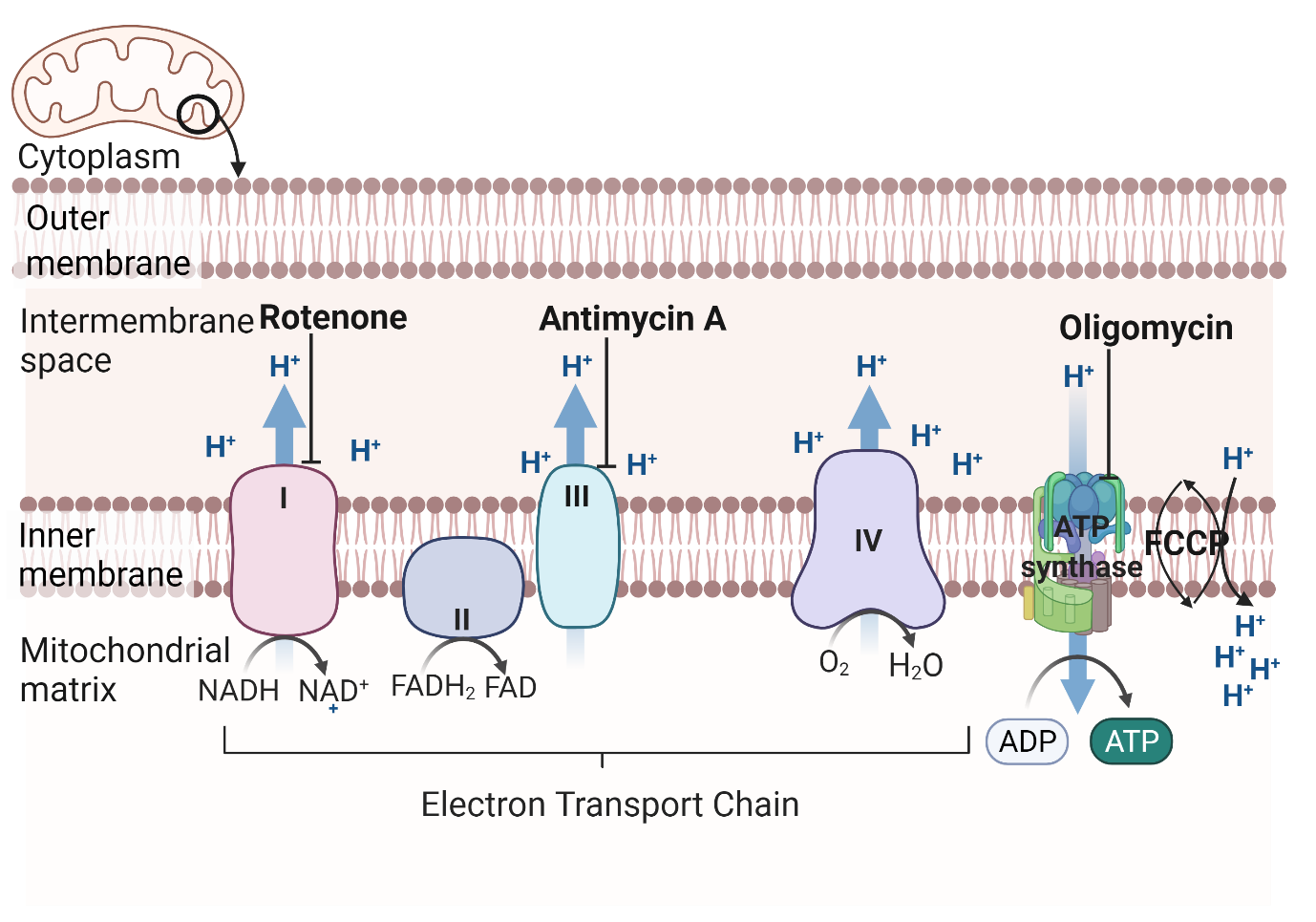


**Supplementary Figure 4. Schematic diagram illustrating the targets of oligomycin, carbonyl cyanide-4-(trifluoromethoxy) phenylhydrazone (FCCP) and Rotenone/Antimycin A on the specific components of the electron transport chain.** Oligomycin inhibits ATP synthase, providing an indication of the proportion of oxygen used for ATP production. FCCP is a mitochondrial uncoupler that dissipates the proton gradient between the matrix and inner membrane space. Rotenone and antimycin A added together to block complexes I and III, respectively shutting down the electron transport chain entirely. *Figure generated using Biorender.*
