# Supplementary material for: Optical imaging detects metabolic signatures associated with oocyte quality
Source: Biol Reprod. 2022 Jul 21;107(4):1014–25. doi: 10.1093/biolre/ioac145 (PMC9562116; doi:10.1093/biolre/ioac145)
Supplement: Supp_table_Assessment_of_oocyte_quality_ioac145 [file supp_table_assessment_of_oocyte_quality_ioac145.docx]

# Supplementary Tables

**Supplementary Table 1. Specification of spectral channels with their respective excitation, emission, dichroic mirror wavelengths and laser powers for hyperspectral system.**

| Spectral Channel | Wavelength (nm) | | | Power (µW) | Exposure time (s) |
| --- | --- | --- | --- | --- | --- |
|  | Excitation | Emission | Dichroic mirror |  |  |
| 1 | 348 | 450 - 475 | 442 | 2.80 | 0.2 |
| 2 | 369 | 450 - 475 | 442 | 2.90 | 0.2 |
| 3 | 371 | 450 - 475 | 442 | 2.70 | 1.5 |
| 4 | 376 | 450 - 475 | 442 | 2.90 | 2.0 |
| 5 | 384 | 450 - 475 | 442 | 2.00 | 2.0 |
| 6 | 390 | 450 - 475 | 442 | 3.98 | 0.8 |
| 7 | 394 | 450 - 475 | 442 | 8.40 | 0.1 |
| 8 | 407 | 450 - 475 | 442 | 4.11 | 0.1 |
| 9 | 420 | 450 - 475 | 442 | 8.63 | 0.1 |
| 10 | 423 | 450 - 475 | 442 | 3.29 | 0.9 |
| 11 | 432 | 450 - 475 | 442 | 6.90 | 2.0 |
| 12 | 348 | 532 - 593 | 560 | 2.76 | 2.0 |
| 13 | 369 | 532 - 593 | 560 | 3.10 | 2.0 |
| 14 | 371 | 532 - 593 | 560 | 2.10 | 2.0 |
| 15 | 376 | 532 - 593 | 560 | 0.87 | 2.0 |
| 16 | 384 | 532 - 593 | 560 | 2.54 | 2.0 |
| 17 | 390 | 532 - 593 | 560 | 3.14 | 2.0 |
| 18 | 394 | 532 - 593 | 560 | 2.17 | 2.0 |
| 19 | 407 | 532 - 593 | 560 | 0.90 | 2.0 |
| 20 | 420 | 532 - 593 | 560 | 3.02 | 2.0 |
| 21 | 423 | 532 - 593 | 560 | 4.60 | 2.0 |
| 22 | 432 | 532 - 593 | 560 | 8.52 | 2.0 |
| 23 | 447 | 532 - 593 | 560 | 10.19 | 2.0 |
| 24 | 449 | 532 - 593 | 560 | 6.92 | 2.0 |
| 25 | 471 | 532 - 593 | 560 | 8.45 | 0.01 |
| 26 | 476 | 532 - 593 | 560 | 9.20 | 2.0 |
| 27 | 480 | 532 - 593 | 560 | 4.90 | 2.0 |
| 28 | 499 | 532 - 593 | 560 | 9.73 | 2.0 |
| 29 | 507 | 532 - 593 | 560 | 13.51 | 2.0 |
| 30 | 522 | 532 - 593 | 560 | 14.80 | 2.0 |
| 31 | 531 | 532 - 593 | 560 | 10.21 | 2.0 |
| 32 | 348 | 690 - 715 | 695 | 14.31 | 2.0 |
| 33 | 369 | 690 - 715 | 695 | 8.33 | 2.0 |
| 34 | 371 | 690 - 715 | 695 | 11.89 | 2.0 |
| 35 | 376 | 690 - 715 | 695 | 12.86 | 2.0 |
| 36 | 384 | 690 - 715 | 695 | 3.13 | 2.0 |
| 37 | 390 | 690 - 715 | 695 | 4.84 | 2.0 |
| 38 | 394 | 690 - 715 | 695 | 8.95 | 2.0 |
| 39 | 407 | 690 - 715 | 695 | 10.73 | 2.0 |
| 40 | 420 | 690 - 715 | 695 | 7.26 | 2.0 |

| Spectral Channel | Wavelength (nm) | | | Power (µW) | Exposure time (s) |
| --- | --- | --- | --- | --- | --- |
|  | Excitation | Emission | Dichroic mirror |  |  |
| 41 | 423 | 690 - 715 | 695 | 8.81 | 2.0 |
| 42 | 432 | 690 - 715 | 695 | 9.55 | 2.0 |
| 43 | 447 | 690 - 715 | 695 | 5.30 | 2.0 |
| 44 | 449 | 690 - 715 | 695 | 10.15 | 0.01 |
| 45 | 471 | 690 - 715 | 695 | 14.25 | 2.0 |
| 46 | 476 | 690 - 715 | 695 | 15.49 | 2.0 |
| 47 | 480 | 690 - 715 | 695 | 10.74 | 2.0 |
| 48 | 499 | 690 - 715 | 695 | 14.88 | 2.0 |
| 49 | 507 | 690 - 715 | 695 | 8.62 | 2.0 |
| 50 | 522 | 690 - 715 | 695 | 12.34 | 2.0 |
| 51 | 531 | 690 - 715 | 695 | 13.35 | 2.0 |
| 52 | 563 | 690 - 715 | 695 | 2.47 | 2.0 |
| 53 | 597 | 690 - 715 | 695 | 3.89 | 2.0 |
| 54 | 625 | 690 - 715 | 695 | 7.15 | 2.0 |
| 55 | 649 | 690 - 715 | 695 | 8.62 | 2.0 |
| 56 | DIC | 532 - 593 | 560 | 5.82 | 2.0 |

**Supplementary Table 2. Results of embryo transfer experiments using embryos derived from imaged or non-imaged COCs**

| **Treatment** | **No. of females (%)** | | | **Live birth rate (%)** | |
| --- | --- | --- | --- | --- | --- |
|  | **Pseudopregnant Recipients** | **Non-pregnant** | **Pregnant (pregnancy rate; %)** | **No. of embryos transferred** | **No of live pups** |
|  | **A** | **B**  **(B/A)** | **C**  **(C/A)** | **D** | **E**  **(E/D)** |
| Non-imaged | 19 | 9 (47.4) | 10 (52.6) | 155 | 68 (43.9) |
| Imaged | 18 | 8 (44.4) | 10 (55.6) | 163 | 74 (45.4) |

Non-pregnant mice were excluded from the calculation of live birth rate. Pregnancy and live birth rate were analyzed by Fisher exact test. No significant differences were found.

**Supplementary Table 3. Individual weight (g) of pups at weaning. Pups derived from COCs that were either imaged or non-imaged using the hyperspectral microscope.**

| Weight of pups at weaning (g) | |
| --- | --- |
| Non-imaged | Imaged |
| 15 | 15 |
| 11 | 13 |
| 13 | 12 |
| 12 | 14 |
| 12 | 15 |
| 13 | 12 |
| 15 | 13 |
| 12 | 9 |
| 12 | 10 |
| 13 | 11 |
| 14 | 13 |
| 13 | 13 |
| 13 | 11 |
| 13 | 14 |
| 9 | 9 |
| 9 | 11 |
| 9 | 11 |
| 11 | 12 |
| 9 | 10 |
| 11 | 10 |
| 9 | 12 |
| 11 | 11 |
| 11 | 12 |
| 9 | 12 |
| 10 | 13 |
| 9 | 12 |
| 11 | 12 |
| 11 | 14 |
| 10 | 10 |
| 13 | 12 |
| 13 | 13 |
| 13 | 12 |
| 14 | 12 |
| 13 | 12 |
| 13 | 10 |
| 13 | 10 |
| 11 | 9 |
| 12 | 10 |
| 10 | 9 |
| 11 | 10 |
| 11 | 9 |
| 12 | 11 |
| 11 | 10 |
| 11 | 10 |
| 10 | 10 |
| 11 | 13 |
| 10 | 10 |
| 12 | 11 |
| 9 | 11 |
| 10 | 12 |
| 11 | 11 |
| 9 | 11 |
| 9 | 11 |
| 11 | 11 |
| 12 | 10 |
| 11 | 13 |
| 12 | 13 |
| 11 | 13 |
| 10 | 13 |
| 10 | 12 |
| 11 | 13 |
| 11 | 12 |
| 10 | 11 |
| 11 | 12 |
| 11 | 12 |
| 11 | 11 |
| 9 | 11 |
| 11 | 11 |
|  | 12 |
|  | 10 |
|  | 10 |
|  | 10 |
|  | 11 |
|  | 10 |
